# Ceragenin Nanogel Coating Prevents Biofilms Formation on Urinary Catheters

**DOI:** 10.1021/acsami.5c13979

**Published:** 2025-07-22

**Authors:** Antonio Puertas-Segura, Antonio Laganá, Garima Rathee, Paul Savage, Katerina Todorova, Petar Dimitrov, Iva Pashkuleva, Rui Luís Reis, Gianluca Ciardelli, Tzanko Tzanov

**Affiliations:** † Grup de Biotecnologia Molecular i Industrial, Department of Chemical Engineering, 16767 Universitat Politècnica de Catalunya, Terrassa 08222, Spain; ‡ Department of Biomedical and Dental Sciences and Morphofunctional Imaging, 18980 University of Messina, Messina 98122, Italy; § Department of Chemistry and Biochemistry, Brigham Young University, Provo, Utah 84606, United States; ∥ Institute of Experimental Morphology, Pathology and Anthropology with Museum, 441043 Bulgarian Academy of Sciences, Geo Milev, Sofia 1000, Bulgaria; ⊥ 3B’s Research Group−Biomaterials, Biodegradables and Biomimetics, University of Minho, Headquarters of the European Institute of Excellence on Tissue Engineering and Regenerative Medicine, Barco 4608-017, Portugal; # ICVS/3B’s PT Government Associated Laboratory, Braga 4710-057, Portugal; ¶ Department of Mechanical and Aerospace Engineering, Politecnico di Torino, Torino 10129, Italy

**Keywords:** urinary catheters, ceragenin, nanogels, self-assembly, ultrasound, antimicrobial and antibiofilm coating

## Abstract

Catheter-associated urinary tract infections (CAUTIs) account for 40% of hospital-acquired infections, increasing health risks, patient discomfort, morbidity, and hospitalisation time. Bacterial colonisation may occur both during catheter insertion and prolonged catheterization by microorganisms in the urinary tract, consequently, increasing the bacteriuria risk due to biofilm formation. Nanogels, a class of soft nanocarriers, offer remarkable versatility for developing functional coatings on indwelling medical devices that can efficiently prevent biofilm formation. In this study, we designed an antibacterial and antibiofilm coating by leveraging the ultrasound-assisted assembly and nanogel self-organization on silicone catheters. The nanogel comprising biocompatible gum arabic and poly­(diallyldimethylammonium chloride) was used to encapsulate a synthetic broad-spectrum antimicrobial peptide mimetic ceragenin (CSA-131). A coating from this bioactive nanogel was sonochemically built on the catheters without any prior surface modification. In vitro and in vivo assays showed that the coating provided antimicrobial and antibiofilm activity for up to 7 days of catheterization, next to catheter lubricity. Cytotoxicity assessment confirmed the absence of toxic effects, underscoring the biocompatibility of the coating formulation. These findings highlighted the potential of nanogels, combined with ultrasound technology, as an innovative approach for durable antimicrobial and antibiofilm functionalization of urinary catheters, particularly susceptible to colonisation by microorganisms upon catheterization.

## Graphical abstract

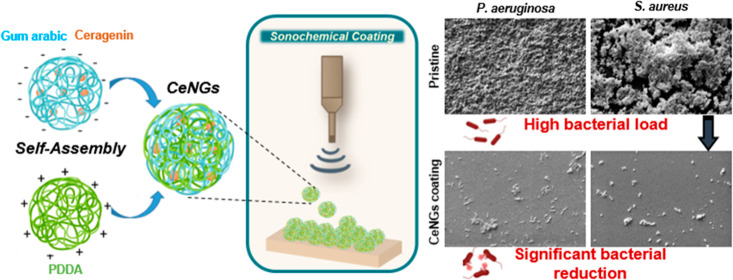

## 1 Introduction

Catheter-associated urinary tract infections (CAUTIs) are the most frequent nosocomial infections with significant impact on public health due to the associated morbidity and mortality. The emergence of antimicrobial-resistant pathogens and resilient biofilms exacerbates these infections, leading to medical device failure, and persistent, hard-to-treat infections.^1^ These complications prolong hospital stays and impose a considerable economic burden on healthcare systems.^2^ So far, the advancements in catheter materials, such as replacement of latex with silicone derivatives to reduce bacterial adhesion, have demonstrated limited efficacy in preventing CAUTIs.^3^ Therefore, new approaches are urgently needed to minimize bacterial colonisation on catheters and lower the risk of device-associated infections.

Nanogels (NGs) have emerged as attractive candidates for the surface functionalization of urinary catheters, providing a platform for effective antimicrobial coatings. NGs are promising materials for building durable, biocompatible, and antibiotic-free coatings on indwelling medical devices prone to bacterial colonisation.^4^ NGs integrate the advantages of nanoparticles and hydrogels: they are characterized by high surface-to-volume ratio and are responsive to environmental stimuli by undergoing volume phase transitions, i.e., swelling.^5^ Unlike bulk hydrogels, the modular assembly of NGs allows for a homogeneous distribution of bioactive components in the three-dimensional networks, enhancing the encapsulation efficiency, stability, and controlled release.^6,7^ Different natural (e.g., polysaccharides) and synthetic polymers have been used for NGs development. Among these, the biobased gum arabic (GA) and the synthetic poly­(diallyldimethylammonium chloride) (PDDA) have the advantage of exhibiting opposite charges that facilitate their self-assembly via polyelectrolyte complexation,^8^ eliminating the need for chemical cross-linking agents and curing steps.^9^ This eco-friendly approach has been applied for coating medical devices due to the associated biocompatibility and cost-effectiveness as well as the strong adhesive properties of the generated polyelectrolyte complexes.^10,11^ Additionally, these complexes can serve as carriers for bioactive molecules such as antimicrobial peptides (AMPs), further augmenting their biofunctionality.

Ceragenins (Ce), a novel class of synthetic antimicrobial agents, mimic the mechanism of action of AMPs, but outperform them in terms of stability upon application and scalable production.^12,13^ These compounds exhibit potent antimicrobial activity against multidrug-resistant pathogens while maintaining excellent biocompatibility.^14^ We hypothesized that the encapsulation of Ce in stimuli-responsive NGs will allow for their controlled in situ release, providing a nanoenabling platform for preventing biofilm-associated infections. To ensure efficient deposition and durability of NGs on catheter surfaces, together with preservation of the intrinsic properties of the device material, ultrasound-assisted coating technology can be employed. This waterborne technology is characterized by fast processing rate, cost-effectiveness, absence of harmful byproducts, and easy upscaling.^15^ Previous studies have shown that the cavitation effect caused by ultrasound in liquids generates high-energy shockwaves, which deposit the NGs on the silicone surface, forming a stable, functional hydrogel coating without the need of physicochemical pre- and post-treatments of the substrate.^16^

In this study, we developed a novel antimicrobial ceragenin-enabled NGs (CeNGs) coating on urinary catheters (Figure 1). High-intensity ultrasound was applied for the CeNGs deposition on silicone catheters, resulting in durable antimicrobial, antibiofilm, and biocompatible coating on these indwelling devices. Moreover, the coating improved surface lubricity, potentially reducing patient discomfort during catheterization. The efficacy of the coating was evaluated both in vitro and in vivo in a rabbit animal model, demonstrating its potential for preventing biofilm formation on medical devices.

**Figure 1:**
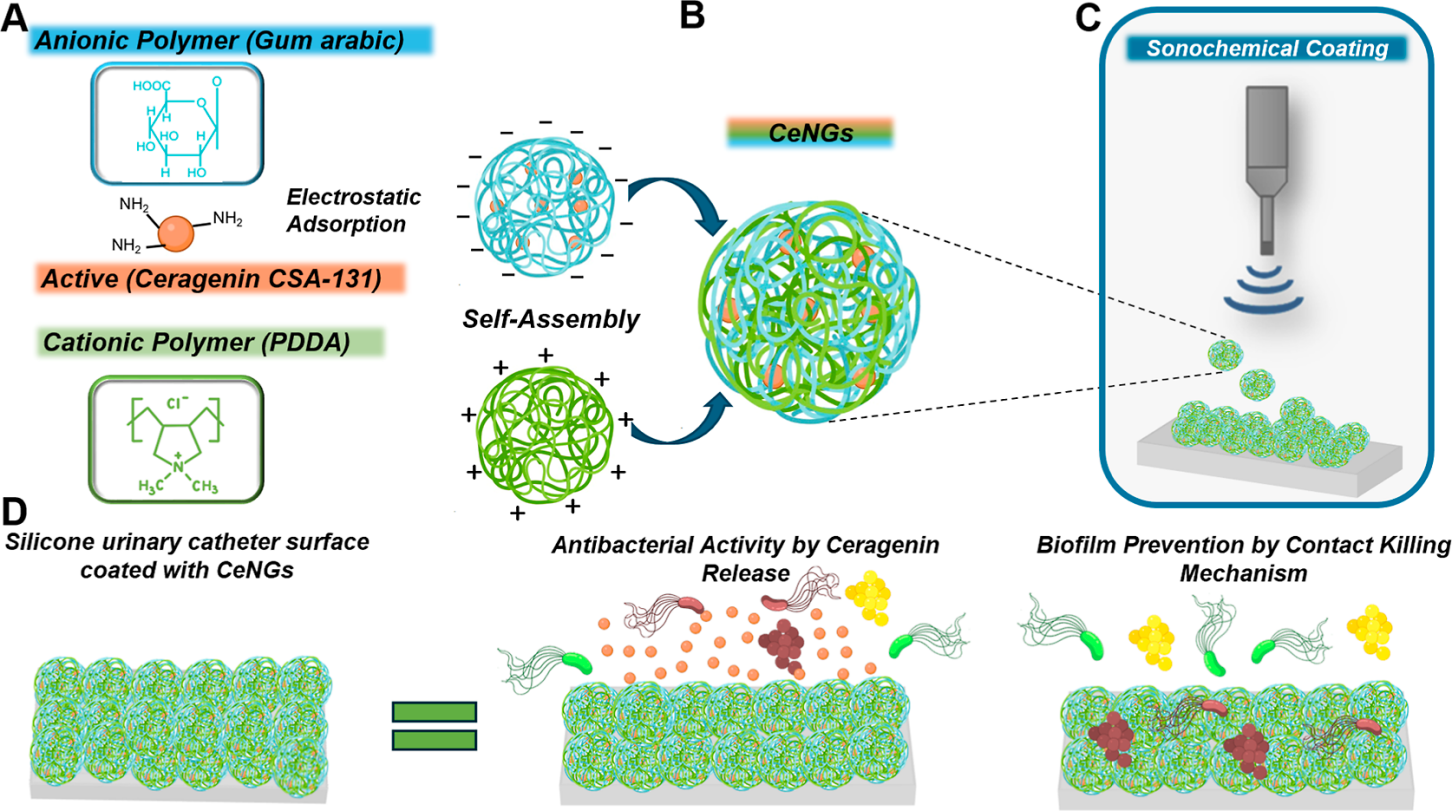
Schematic presentation of the approach for development of CeNGs-based nanoenabled coating for biofilm prevention on urinary catheters: (A) Chemical structures of the used components (essential charged groups for polyelectrolyte complexations are indicated with red arrows); (B) Polyelectrolyte complexation leads to the formation of nanogels (CeNGs); (C) CeNGs coating is formed by ultrasound deposition of the formed complexes; (D) The bactericide effect of the coating is due to Ceragenin (CSA-131) release and contact killing mechanism.

## 2 Experimental Section

### 2.1 Materials

Polydimethyl/vinylmethyl siloxane (PDMS), classified as VMQ polymer according to ASTM D1418, was supplied by Degania Silicone Ltd. (Israel) and used in the form of urinary Foley catheters and flat sheets. Ceragenin CSA-131 was synthesized via a cholic acid scaffolding approach, as previously reported.^17^ GA, PDDA, acetone, Muller-Hinton broth (MHB), tryptic soy broth (TSB), Luria–Bertani broth (LB), sodium dodecyl sulfate (SDS), phosphate-buffered saline (PBS), cetrimide agar, Baird-Parker agar, nutrient broth agar, resazurin salt, crystal violet, Dulbecco’s modified Eagle’s medium (DMEM), *N*-acetyl homoserine lactone (AHL) and the Proliferation Kit II XTT were all purchased from Sigma-Aldrich. The ethanol used was from Scharlab (Spain). The Live/Dead BacLight bacterial viability kit (Molecular Probes L7012) and the AlamarBlue cell viability reagent were obtained from Invitrogen.

Microbial strains () (ATCC 25923) and () (ATCC 10145) (CECT795) () and () (ATCC 25922), as well as, human fibroblast (ATCC-CRL-4001, BJ-5ta) and keratinocyte (HaCaT) cells were sourced from the American Type Culture Collection (ATCC LGC Standards). CECT 5999 ( CV026) was obtained from the Spanish Type Culture Collection (CECT, Spain).

### 2.2 Synthesis and Characterization of CeNGs

NGs were synthesized by dissolving 20 mg of CSA-131 in 20 mL GA (50 mg/mL) aqueous solution. Subsequently, 10 mL 5% (w/v) solution of PDDA in water was added dropwise at a 0.5 mL/min rate under continuous stirring. To facilitate CeNG precipitation and remove the nonreacted compounds, the obtained dispersion was treated with acetone at a sample-to-solvent ratio of 1:10, followed by resuspension in 30 mL deionized water. The final NG suspension was stored at 4 °C until further use.

The formation of NGs and their size were confirmed by scanning electron microscopy (SEM) using a Helios 450S (FEI). The surface charge was determined by Zetasizer Nano Z (Malvern). The quantification of CSA-131 was carried out by measuring amino content through fluorescamine assays.^18^ The biocidal activity of the NGs without ceragenin (WNGs) and CeNGs was evaluated against and through standard dilution assays. Following treatment with NGs, bacterial metabolic activity was assessed using a resazurin-based viability assay.^19^ The antibiofilm properties of the NGs were determined by quantifying biomass reduction using crystal violet staining as a colorimetric indicator.^20^ Additionally, the effect of NGs on the bacterial quorum sensing (QS) was investigated using as a model organism.^21^

### 2.3 Silicone Coating with CeNGs

PDMS specimens (1 × 1 cm, *n* = 10) were excised and sequentially washed with 0.1% (w/v) sodium dodecyl sulfate (SDS) solution, deionized water, and 96% ethanol to remove any surface contaminants. The cleaned specimens were then immersed in 30 mL of the NGs suspension and subjected to ultrasound at 50% amplitude generated with an ultrasonic processor (VCX 750, Sonics & Materials) for 30 min at 25 °C. Following sonication, the samples were removed from the reaction vessel and rinsed thoroughly with deionized water to eliminate the loosely fixed NG material. The coated segments were dried under a nitrogen stream and stored at 4 °C until further analysis. Additionally, urinary catheters were coated by immersing them in 100 mL of the same NG suspension under identical ultrasonic conditions.

### 2.4 Characterization of the Coating

Attenuated Total Reflectance-Fourier Transformed infrared (ATR-FTIR) spectra of both pristine and modified silicone samples were recorded over the 650–4000 cm^–1^ range using a PerkinElmer Spectrum 100 FTIR spectrometer. Each spectrum was obtained by averaging 64 scans at a resolution of 4 cm^–1^.

The chemical composition of the coatings was analyzed using X-ray photoelectron spectroscopy (XPS) with a SPECS Surface Nano Analysis system (SPECS Surface Nano Analysis GmbH), equipped with a high-intensity twin-anode X-ray source XR50 (Mg/Al at 1253 eV/1487 eV) operating at 150 W. The X-ray source was positioned perpendicular to the analyzer axis, and a Phoibos 150 MCD-9 XP detector was utilized. Survey and narrow scans were conducted with pass energies of 25 and 0.1 eV, respectively. Charge compensation was achieved using a combination of electron and argon ion flood guns, with electron energy and emission currents set to 4 eV/0.35 mA and 0 eV/0.1 mA, respectively. Spectra acquisition was performed at a pass energy of 25 eV in 0.1 eV steps under ultrahigh vacuum conditions (pressure <6 × 10^–9^ mbar). Standard charge compensation parameters ensured a uniform static charge across the samples. The C 1s peak at a binding energy of 285 eV was used as an internal reference.

SEM was utilized to analyze the surface morphology of the silicone catheter surface after coating. Before imaging, the samples were coated with a 5–10 nm thick conductive layer of gold/platinum to ensure optimal imaging conditions. The imaging parameters, including accelerating voltage and working distance, were adjusted to optimize the resolution and contrast. High-resolution micrographs were acquired at different magnifications to evaluate the coating’s uniformity.

The surface roughness of both uncoated and coated samples were analyzed using atomic force microscopy (AFM) (Dimension 3100 AFM system by Veeco) operating in tapping mode. The resulting AFM images were subsequently processed using Nanotec WSxM software for detailed analysis.^22^

The water contact angle (WCA) of pristine, WNGs and CeNGs-coating samples were measured using a contact angle analyzer (DSA 25 Krüss) with data acquisition and analysis performed by Krüss Advanced v1.13.0.21301 software. Measurements were conducted using the sessile drop method, and the contact angle was determined by applying the tangential method for precise calculation of WCA.

### 2.5 Release Kinetic of Ceragenin from the Coatings

The release of CSA-131 was evaluated by immersing coated 1 × 1 cm PDMS samples in 2 mL sterile PBS and incubating them in a water bath at 37 °C with agitation at 100 rpm. At 24 h intervals, aliquots (100 μL) were removed, and the release medium was replaced with fresh sterile PBS to maintain sink conditions. The aliquots were analyzed by liquid chromatography–mass spectrometry (LC/MS) to quantifying the released CSA-131. A mass-labeled internal standard (CSA-131D_25_) was employed for accurate quantification, with analysis conducted on a 6230 TOF LC/MS system (Agilent Technologies).

### 2.6 Coating Lubricity

The frictional behavior of both coated and uncoated silicone samples (8 × 20 cm) was assessed using a ZwickRoell universal tribometer (ZwickRoell GmbH & Co). In the setup, the silicone specimen was secured to the stationary base, while the baize fabric was fixed to the mobile arm. A normal load of 0.2 N was applied during testing. The sliding motion was linear, covering a distance of 50 mm at a rate of 10 mm/s. The tangential force exerted during the test was monitored continuously through the TestXpert III software. Two types of friction coefficients were calculated: the static coefficient of friction (μ_S_), derived from the peak force registered before the onset of movement, and the dynamic coefficient of friction (μ_D_), based on the mean force observed during sliding.

### 2.7 Antibiofilm Properties of the NG Coatings

#### 2.7.1 Antimicrobial Activity toward Planktonic Bacteria

The antimicrobial activity of the coated PDMS samples (1 × 1 cm) was evaluated following the ASTM-E2149–20 standard. Individual colonies of and were cultured overnight in 5 mL sterile MHB at 37 °C with agitation at 230 rpm. The bacterial cultures were then diluted in PBS (OD_600_ = 0.28). A further 1:1000 dilution was performed in PBS, and each PDMS sample was incubated with 1.5 mL of the diluted bacterial suspension. After 24 h of incubation, the suspensions were collected, and the number of viable bacteria was determined by plating aliquots onto cetrimide agar for and Baird-Parker agar for . Colony-forming units (CFU) were quantified to assess bacterial survival and determine the antimicrobial efficacy of the coated PDMS samples.

#### 2.7.2 Quantification of the Biofilm Biomass

The inhibition of biofilm formation was assessed by quantifying the biomass of and biofilms. To this end, PDMS samples (1 × 1 cm) were incubated with 1.5 mL bacterial suspension (OD_600_ = 0.01) in TSB, placed in a 24-well microplate and maintained under static conditions at 37 °C for 24 h to allow bacterial adhesion and biofilm development. Following incubation, the samples were washed three times with 2 mL sterile 100 mM PBS, pH 7.4, to remove planktonic bacteria. The adhered biofilms were then fixed by drying at 60 °C for 2 h. To quantify the biofilm biomass, the fixed biofilms were stained with 1 mL 0.1% (w/v) crystal violet solution for 15 min. The excess stain was removed by rinsing with 1 mL 30% (v/v) acetic acid to solubilize the bound crystal violet. A 100 μL aliquot from each sample was transferred to a 96-well microplate, and its absorbance was measured at 595 nm to determine biofilm formation.

#### 2.7.3 Bacterial Viability in the Biofilm

The metabolic activity of bacterial cells in the biofilm formed on PDMS surfaces was evaluated using the Cell Proliferation Kit II (XTT). PDMS samples (1 × 1 cm) were incubated with 1.5 mL TSB bacterial suspension (OD_600_ = 0.01) in a 24-well microplate and maintained at 37 °C under static conditions for 24 h to allow biofilm formation. Following incubation, the samples were treated with 1 mL of the preprepared XTT kit solution, which consisted of XTT and phenazine methosulfate (PMS). The samples were then incubated for an additional 2 h at 37 °C to allow metabolic conversion of the XTT reagent. After incubation, the absorbance was measured at 492 and 690 nm using a microplate reader to quantify bacterial viability within the biofilm.

#### 2.7.4 Morphology of the Biofilm via Fluorescence Microscopy and SEM

The structural organization and bacteria viability in biofilms formed on PDMS surfaces were assessed using fluorescence microscopy and SEM. The biofilms were stained with Live/Dead BacLight bacterial viability kit for fluorescence microscopy to differentiate live from dead bacterial cells. Micrographs were captured to analyze the biofilm distribution qualitatively. Viability was determined based on fluorescence emission: Syto 9 (λ_ex_ = 480 nm, λ_em_ = 500 nm) stains live cells, whereas propidium iodide (λ_ex_ = 490 nm, λ_em_ = 635 nm) selectively penetrates and stains dead cells, providing insight into bacterial viability within the biofilm. To investigate the topographical features of the coatings and their influence on and biofilms, PDMS samples (1 × 1 cm) were incubated with 1.5 mL of bacterial suspension (OD_600_ = 0.01) in TSB for 24 h at 37 °C under static conditions. Biofilms were then fixed by incubation in a 2% paraformaldehyde and 2.5% glutaraldehyde solution overnight. Samples were dehydrated through a graded ethanol series (25%, 50%, 75%, and 100%) for 30 min at each step. Due to the nonconductive nature of silicone, the samples were coated with a thin layer of gold/platinum prior to imaging. Biofilm morphology and surface interactions were then analyzed using SEM (EVO MA10, Zeiss, Germany).

#### 2.7.5 Biofilm Formation under Dynamic Conditions

An in vitro model simulating a catheterized human bladder was employed to assess the stability of the coating and its efficacy in preventing biofilm formation under dynamic conditions.^23^ Both uncoated and coated silicone Foley catheters were introduced into the sterile model, with the catheter balloon inflated using 5 mL of 100 mM sterile PBS. The bladder model was then filled to the catheter eye with sterile artificial urine (pH 6.8), prepared according to UNE EN1616 (Sterile Urethral Catheters for Single Use), and supplemented , , , and at a final concentration of 1 mg/mL TSB (OD_600_ = 0.01).

The system was maintained at 37 °C for 7 days with a continuous flow of artificial urine at a 1 mL/min rate to mimic physiological conditions. At the end of the experiment, the catheters were removed, and biofilm formation in the lumen and external surface of the catheter was evaluated by fluorescence microscopy. Urinary catheters were longitudinally sectioned and washed three times with deionized water to remove the nonadhered bacteria. Both the inner and outer surfaces were stained using the Live/Dead kit, following the protocol detailed in Section 2.7.4.

### 2.8 In vitro Assessment of Coating Biocompatibility

As a preliminary step to assess the safety of the developed coatings, in vitro biocompatibility tests were conducted following the international standard ISO 10993–5. Fibroblasts (BJ-5ta) and keratinocytes (HaCaT) were preseeded at a density of 4.5 × 10^4^ cells per well in a sterile 24-well tissue culture-treated polystyrene plate. For cytotoxicity evaluation, pristine and coated PDMS samples (1 × 1 cm) were placed in direct contact with the cells. Subsequently, 0.75 mL of complete growth medium (DMEM) was added to each well, and the plate was incubated at 37 °C in a humidified atmosphere with 5% CO_2_ for 24 h.

Cell viability was assessed using the AlamarBlue assay kit, following the manufacturer’s instructions. Cells cultured under the same conditions but without PDMS samples were used as negative controls. Fibroblast and keratinocyte viability was also evaluated using the Live/Dead Viability/Cytotoxicity kit, according to the supplier’s protocol. Fluorescence microscopy was employed to visualize live and dead cells, providing qualitative confirmation of biocompatibility.

### 2.9 In vivo Validation of the Coated Catheters in a Rabbit Model

New Zealand male rabbits (4 months old, weighing 3.5–4 kg) were used to assess the biocompatibility of the coating. The animals were housed in individual cages with ad libitum access to food and water. All experimental procedures were conducted in compliance with national regulations on laboratory animals and animal welfare (No. 20/01.11.2012), as well as Directive 2010/63/EU of the European Parliament, and were approved by the Ethical Committee of the Institute of Experimental Morphology, Pathology, and Anthropology with Museum, Bulgaria (No. 282/24.09.2020). Following a quarantine period of 2 weeks, the rabbits underwent a medical examination and were divided in two groups: the control group (*n* = 3) was catheterized using unmodified silicone Foley catheters (French size 8), while the experimental group (*n* = 3) received coated Foley catheters. The dwell time for both groups was 10 days. Catheter insertion was performed under general anesthesia, administered as a combination of tiletamine/zolazepam, xylazine, and butorphanol at 5, 4, and 0.15 mg/kg body weight, respectively.

Before insertion, both treated and untreated catheters were sterilized under UV light for 15 min. The catheterization procedure was performed under aseptic conditions and guided via ultrasound imaging (Mindray DP-20 Vet). Following insertion into the bladder, the external segment of the catheter was trimmed to 0.2–0.3 cm within the urethral orifice, and the catheters were secured to the preputial skin using multiple dermal sutures. Standard disinfection protocols were observed throughout the procedure, and the rabbits were fitted with cervical collars to prevent interference with the implanted devices. All animals exhibited full recovery postcatheterization and were subjected to daily medical evaluations throughout the study. Blood samples were collected from the jugular vein both before the beginning and upon completion of the survey. Fresh sterile urine samples (5 mL) were collected from both groups on two separate occasions in sterile urine containers. Additionally, immediately after euthanasia, sterile urine was obtained both through the catheters and directly from the bladder. Fresh sterile urine samples (10 mL) and catheter segments taken from the central portion of the devices were also aseptically obtained.^24^ Urine samples underwent urinalysis using a urine analyzer with test strips (Urit 50 Vet). Complete blood counts and biochemical analyses were performed using an automated hematology analyzer (Mindray BC 2800 Vet) and a blood chemistry analyzer (MNCHIP Celercare V2). All samples were subsequently submitted to microbiological evaluation in the medical diagnostic laboratory “RAMUS” in Sofia, Bulgaria. Histological samples were collected from the urethra, bladder, and kidneys. These tissues were fixed in 10% formalin, dehydrated, cleared in xylene, and embedded in paraffin. Sections of 3–5 μm thickness were stained with hematoxylin and eosin and examined under a Leica DM 5000B microscope for lesions and signs of inflammation or infection.

### 2.10 Statistical Analysis

All reported values are expressed as the mean ± standard deviation. For multiple comparisons, statistical analyses were conducted using GraphPad Prism Software 5.04. A one-way analysis of variance (ANOVA) was employed, followed by Tukey’s post hoc test, or an unpaired two-tailed Student’s *t*-test was applied where appropriate. Statistical significance was set at *p* < 0.05 (*), *p* < 0.01 (**), and *p* < 0.001 (***).

## 3 Results and Discussion

### 3.1 Synthesis of CeNGs

CeNGs were obtained through polyelectrolyte complexion between the oppositely charged macromolecules (Figure 1a) GA with ζ-potential of −30 mV and PDDA with ζ-potential of +40 mV. The spontaneously generated nanometric hydrogel network effectively encapsulated 54% of CSA-131 under ambient conditions. SEM analysis revealed an average size of approximately 100 nm for the NGs with or without CSA-131 (Figure 2A). The NG alone had limited antimicrobial activity: they were active against at high concentrations but could not inhibit the growth of (Figure 2B) at any of the assessed concentrations. On the contrary, the loaded CeNGs demonstrated significantly higher antimicrobial activity against and at concentrations below 2% (Figure 2B) The enhanced antibacterial effect of NGs against planktonic compared to may be attributed to structural differences in their cell envelopes. As a Gram-positive bacterium, lacks the outer membrane present in Gram-negative , which acts as a permeability barrier and limits the interaction with cationic agents and their internalization.^25^ NGs ability to disrupt QS was assessed using the bacterial model , because it produces the purple pigment violacein in the presence of *N*-acyl-homoserine lactone signaling molecules involved in QS (Figure 2C). Both NG compositions reduced the violacein production indicating inhibition of the bacterial communication pathways, essential for early stage biofilm formation.^26^ This effect may be attributed to the presence of gum arabic that can interfere with QS by inhibiting the autoinducer synthesis or by sequestering the signaling molecules, thereby impairing their ability to activate QS-regulated responses.^27,28^ The biofilm-preventing capabilities of the NGs were further confirmed through quantitative assays against and (Figure 2D). CeNGs exhibited superior inhibition of biofilm biomass compared to WNGs, even at concentrations as low as 0.06%, highlighting the synergistic effect between the antimicrobial action of ceragenin and the suppression of QS signals. Altogether these results demonstrate that CeNGs exert their action via multiple complementary mechanisms, including direct bactericidal activity and microbial communication disruption, offering a robust strategy for preventing biofilm formation and related bacterial infection.

**Figure 2:**
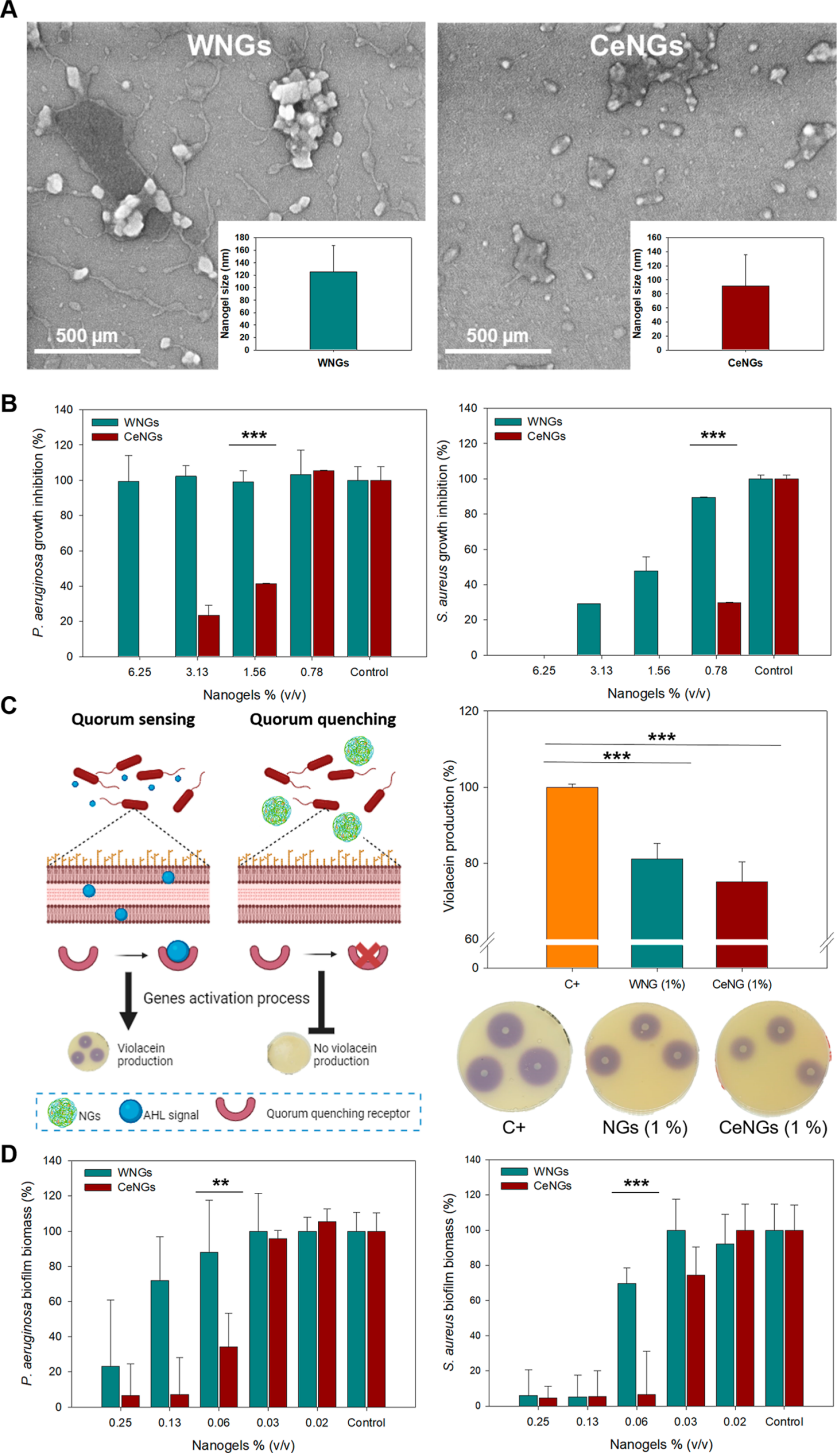
(A) SEM micrographs of nanogels without (WNGs) and with ceragenin (CeNGs). (B) Antimicrobial activity of WNGs and CeNGs toward and . (C) Quorum sensing inhibition in by NGs and CeNGs. (D) and biofilm prevention by WNGs and CeNGs.

### 3.2 Coating of Silicone Catheters with NGs

Conventional catheter coating often requires surface pretreatment, long processing time, or harsh chemical conditions. In contrast, the NGs coatings were developed on urinary catheters using a one-step waterborne sonochemical process, taking only 30 min and without prior modification of the catheter surface for coating adhesion. The NGs’ deposition on the silicone substrate under high-intensity ultrasound is primarily driven by cavitation dynamics and electrostatic macromolecular interactions. The ultrasound generates microjets and shock waves due to the collapse of cavitation bubbles, which propel in a “throwing stone” mode the NGs at high velocity toward the hydrophobic silicone surface, building the coating.^24^ Different physicochemical interactions mediate the adhesion of NGs on the silicone surface. The hydrophobic PDMS does not favor the adhesion of hydrophilic molecules such as GA, which contains hydroxyl groups capable of forming hydrogen bonds with water.^29^ This ability of GA to interact with water molecules in the vicinity of the silicone surface allows to overcome the hydrophobic repulsion, resulting in an initial adhesion of the NGs. By generating cavitation and pressure waves, ultrasound reduces the water exclusion layer (depletion layer) that typically forms on hydrophobic surfaces, allowing the NGs to approach the silicone surface closely and adhere to it. Cavitation expels the water from the interface between NGs and silicone, promoting the transition from hydrated repulsion to dry adhesion.^30^ The deposition of preformed NGs on medical devices offers superior surface coverage, structural uniformity, and mechanical resilience compared to conventional bulk hydrogel coatings.^31^

The NGs deposition on the catheter surface was confirmed by FTIR and XPS (Figure 3A,B). The FTIR spectra of the coated substrates (both WNGs and CeNGs) showed new signals at 3000–3500 cm^–1^ for the hydroxyl groups from GA^32^ and at 1650 cm^–1^ for quaternary amino groups of PDDA.^33^ XPS spectra corroborated these data: the signal at 287.5 eV in the C 1s spectrum of the coated samples is assigned to C–OH bonds and provides clear evidence of GA deposition onto the silicone surface.^34^ Further evidence for the coating deposition was the signal at 401.5 eV in N 1s assigned to charged nitrogen (N^+^).^35^ The presence of ceragenin in the CeNGs coating was confirmed by XPS: lower-energy signals (BE = 398 and 397 eV, Figure 3C) characteristic of primary and secondary amine groups were detected in the N 1s spectrum.^36^

**Figure 3:**
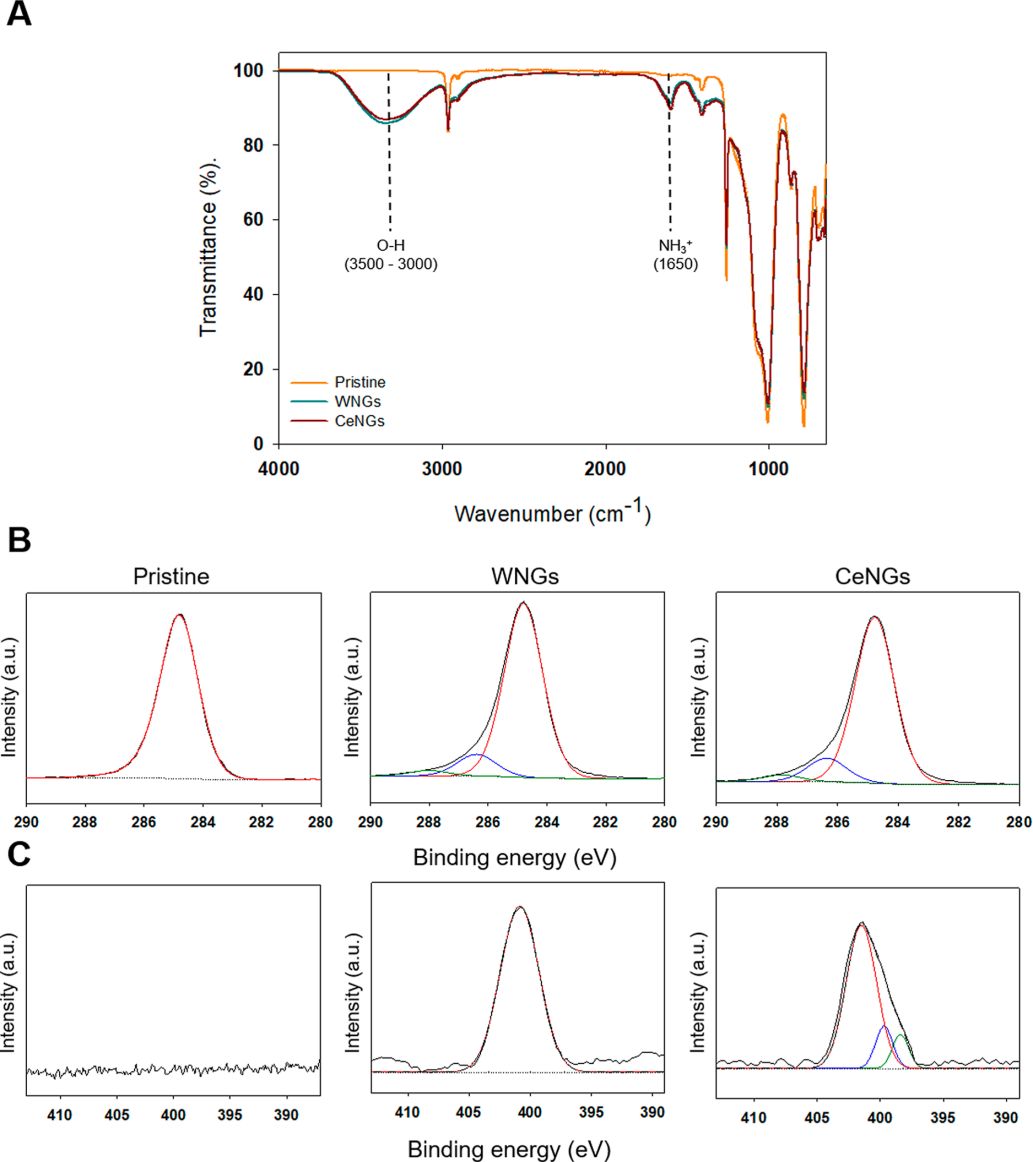
(A) FTIR-ATR spectra of pristine PDMS (Pristine), NGs coating (WNGs), and CeNGs coatings. (B) High-resolution XPS spectra of C 1s, (C) high-resolution XPS spectra of N 1s.

The surface morphology of pristine and NGs-coated substrates was studied by SEM. The surface of the pristine samples was smooth, lacking any additional topographical features. The morphology of the coated samples was different (Figure 4A): NGs were uniformly distributed on the surface, organized in a continuous and homogeneous layer. AFM provided quantitative data about these morphological changes (Figure 4B). Pristine silicone exhibited a low average roughness (R_a_) of 4 nm, while the WNGs coating displayed a discrete morphological features with a substantial increase in roughness, reaching a *R*_a_ of approximately 65 nm. Unlike WNGs, CeNGs merged within more uniform layer with a *R*_a_ of 50 nm. The reduced R_a_ observed on the CeNGs-coated silicones suggests that ceragenin enhances cohesion among the nanogels, promoting a continuous surface coverage. Notably, the coating roughness variation complies with the urinary catheter manufacturing standards, ensuring compatibility with existing production processes.^37^

**Figure 4:**
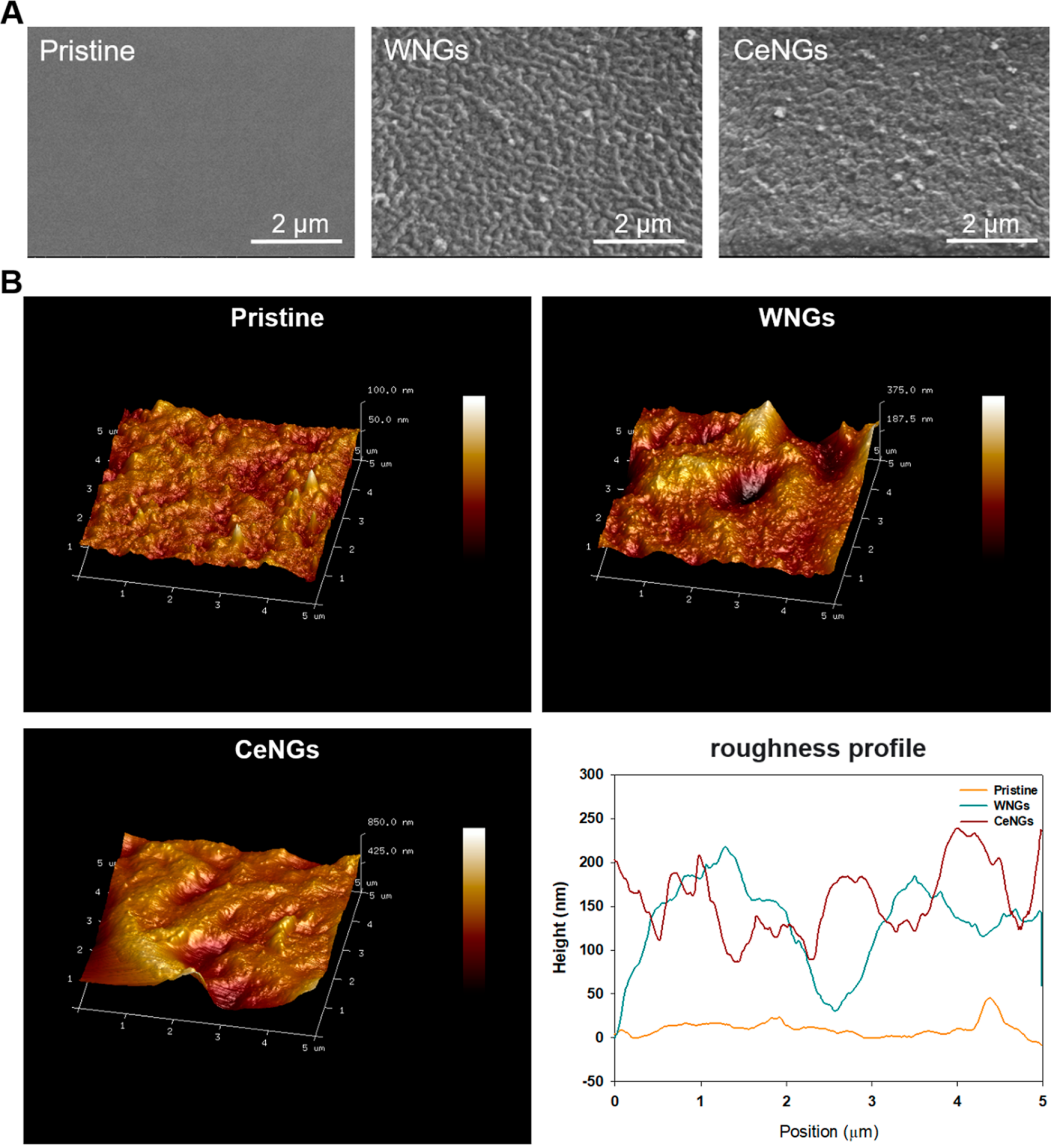
(A) SEM micrographs of pristine and coated substrates. (B)­Three-dimensional AFM images of untreated silicone (Pristine), coated with NGs without ceragenin (WNGs) and ceragenin-loaded NGs (CeNGs). Roughness profile of the samples.

The stability of the NG coating was confirmed by the reduction of the inherent hydrophobicity of the silicone^24^ and the release profile of ceragenin. WCA analysis revealed a significant increase in the material’s hydrophilicity upon coating, that was more pronounced for CeNGs (Figure 5A). After 7 days of immersion in water at 37 °C under agitation, the coating remained hydrophilic. Samples containing ceragenin initially exhibited greater hydrophilicity, which was then restored to values similar to the coatings without ceragenin. Following an initial burst release during the first day, a sustained release of roughly 4–6 μg/mL ceragenin per day was observed for the remaining incubation period (Figure 5B), corresponding to approximately 67% of the total ceragenin content. The sustained release pattern suggests that the coating can deliver ceragenin at least for 7 days ensuring durable antimicrobial and antibiofilm efficacy.

**Figure 5:**
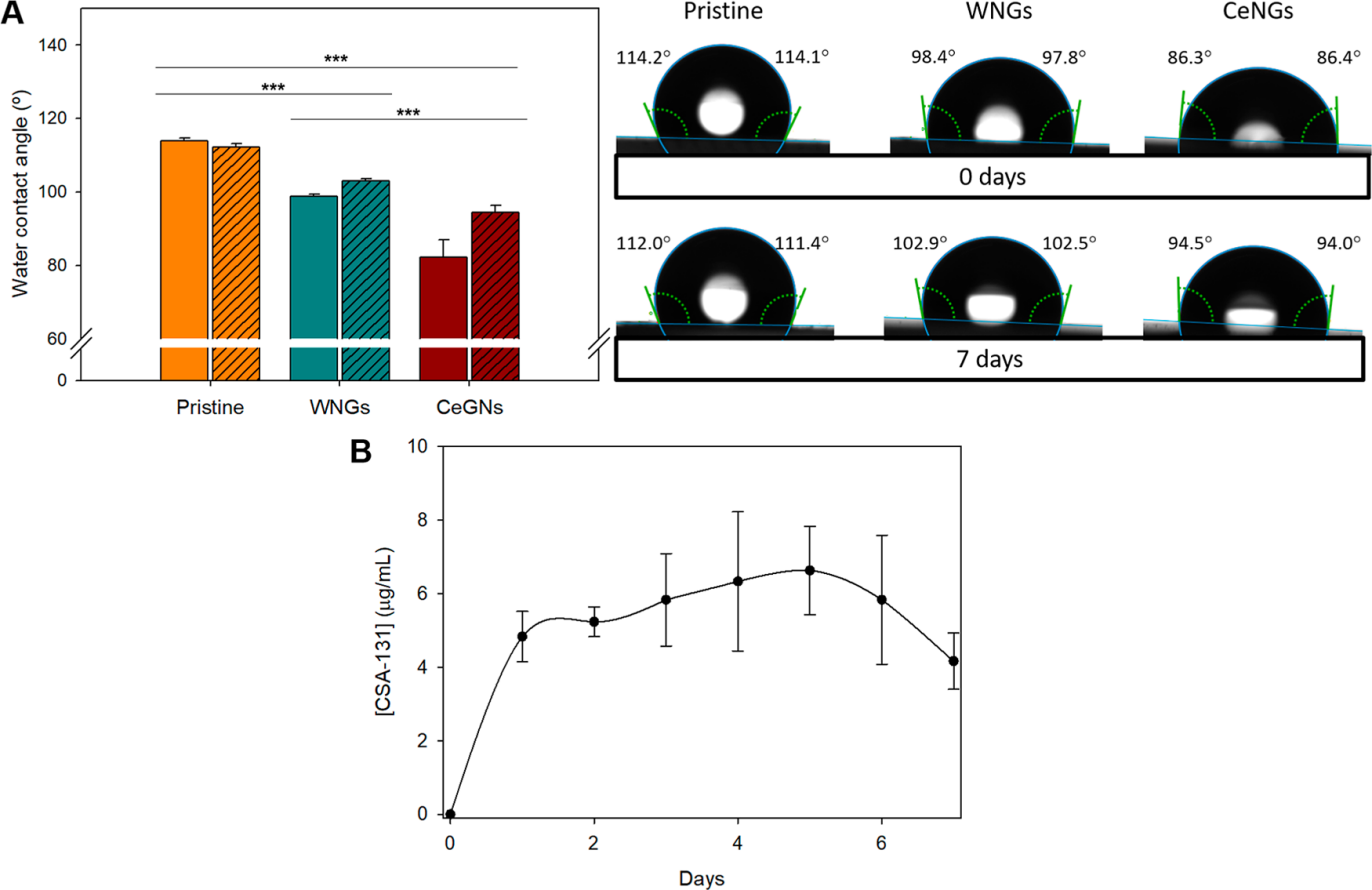
(A) WCA measurements of the NG-enabled coatings before and after 7 days of incubation in deionized water (*n* = 10). Solid-colored bars represent fresh samples (nonincubated), while diagonally hatched bars correspond to samples incubated for 7 days. (B) Release profile of ceragenin in PBS after 7 days at 37 °C (*n* = 3).

### 3.3 Lubricity of the Coating

The application of CeNGs coating significantly improved the lubricity of the silicone substrate, which is crucial for reducing discomfort, epithelial damage, and risks associated with catheterization. Both static and dynamic friction coefficients (CoFs) were assessed to evaluate the frictional resistance to sliding (Figure 6). In the friction–deformation profile (Figure 6A), pristine samples exhibited a sharp increase in friction, reaching CoF values above 1.4 ± 0.05. This behavior is attributed to the silicone viscoelastic nature, where increasing contact area and mechanical hysteresis under deformation amplify the resistance to motion.^38^ In contrast, the CeNGs-coated surface maintained a markedly lower and stable CoF (∼0.8 ± 0.03), indicating reduced surface adhesion during sliding.

**Figure 6:**
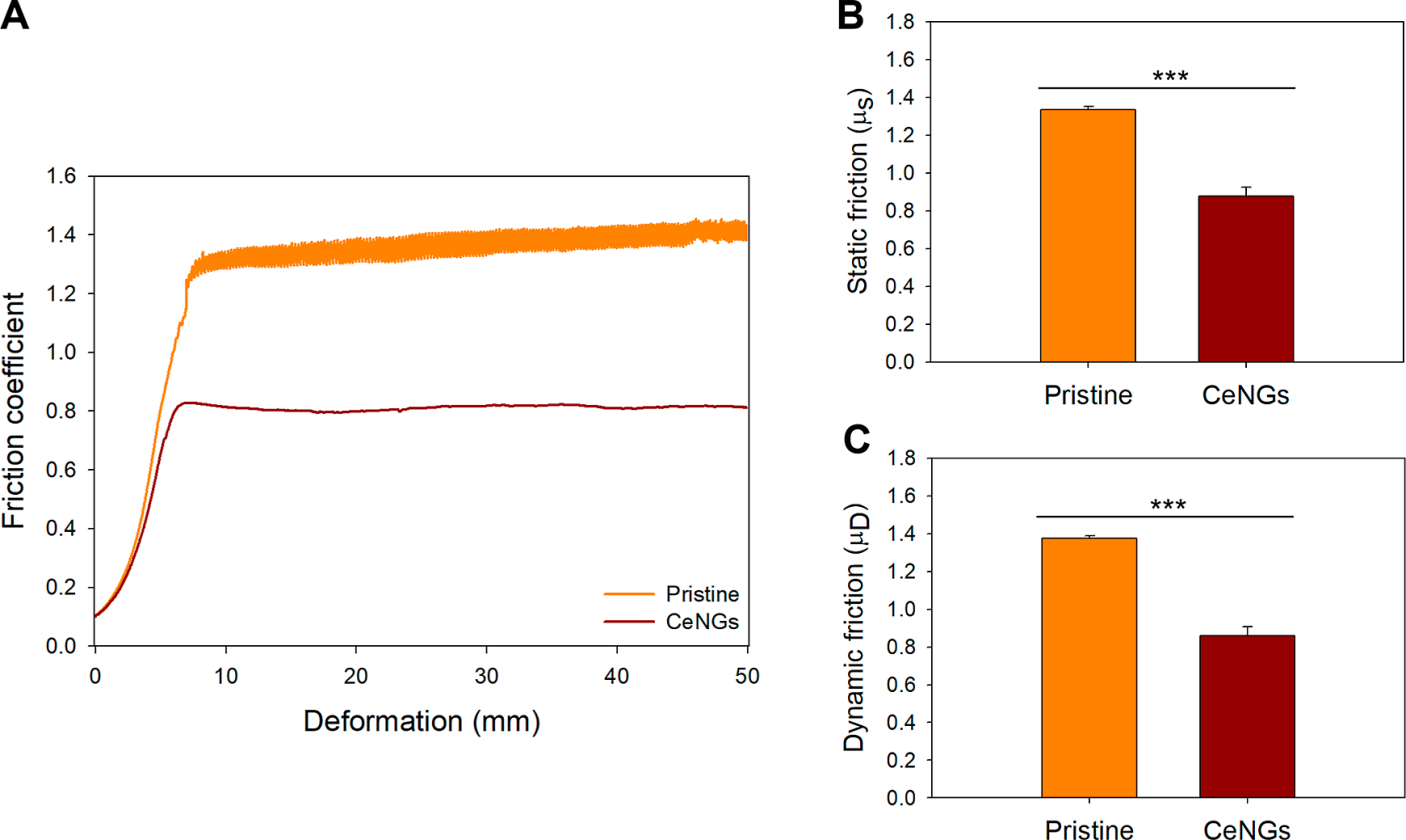
Lubricity of the CeNGs coating (A) CoF study of nontreated and coated silicone (*n* = 3). (B) Static friction. (C) Dynamic friction.

Quantitative analysis of the static CoF (μ_s_, Figure 6B) showed a statistically significant decrease for the CeNGs-coated samples compared to pristine silicone. Likewise, the dynamic CoF (μ_D_, Figure 6C) markedly decreased upon coating confirming the effectiveness of the CeNGs layer in mitigating the sustained mechanical resistance to continuous movement over its surface. Such friction parameters drop suggests the coating’s ability to minimize shear-induced tissue trauma and highlights its potential for improving patient comfort and safety in urinary catheterization procedures.

### 3.4 Antimicrobial Properties

The antimicrobial efficacy of the hybrid coating was evaluated against and , two of the most prevalent uropathogens implicated in urinary tract infections (UTIs). Quaternary ammonium groups in the PDDA macromolecules endow the NGs with intrinsic antimicrobial properties, facilitating bacterial eradication through contact-killing mechanisms.^39^ However, a substantial enhancement in antimicrobial performance was observed for the CeNGs coatings (Figure 7), pointing to a synergistic effect in the eradication of both bacterial species due to contact-mediated nonspecific membrane disruption and sustained active release (Figure 1D). This combined antimicrobial mechanism led to approximately 4-log reduction of the planktonic bacterial viability, compared to the WNGs coating, which only achieved a 2-log reduction, further demonstrating a comparable effect against both Gram-positive and Gram-negative bacteria.

**Figure 7:**
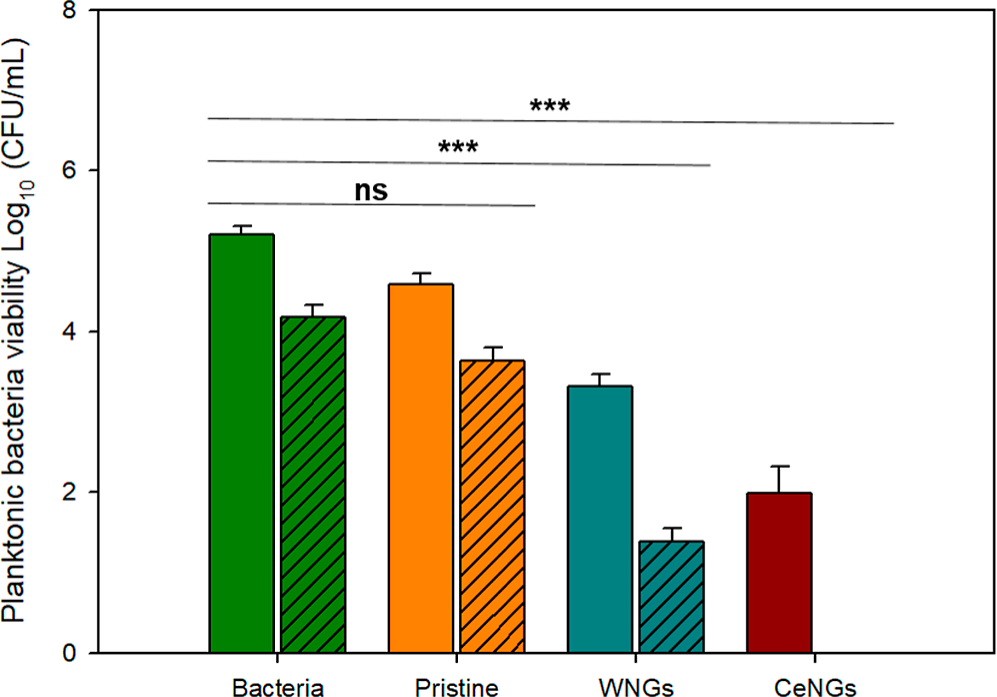
Antibacterial activity of NGs coatings against and (*n* = 3). Solid-colored bars represent and diagonally hatched bars correspond to .

### 3.5 Antibiofilm Properties

The prevention of and biofilm formation was assessed under static conditions by analyzing the bacterial biomass accumulated on the catheter material and the bacterial viability within the biofilm (Figure 8A,B). The experiments were conducted for 24 h to simulate the initial stages of biofilm formation postcatheterization.

**Figure 8:**
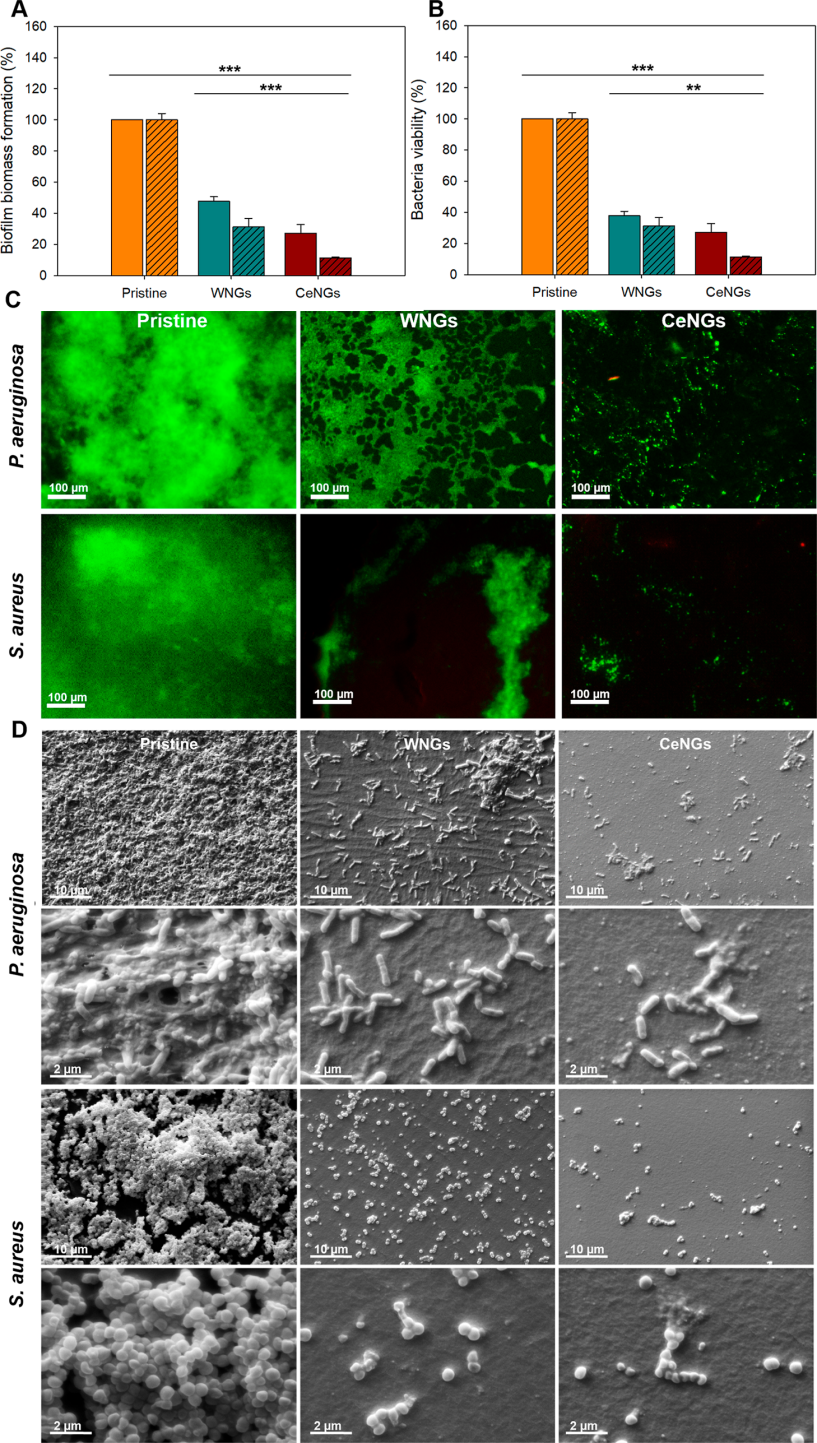
Antibiofilm properties of NGs coatings at static conditions (24 h). (A) Biofilm biomass inhibition. (B) Cell viability of and in biofilms cultured for 24 h on different coatings under static conditions (*n* = 3). Solid-colored bars represent and diagonally hatched bars correspond to . (C) Fluorescence micrographs of live (green) and dead (red) and cells. (D) SEM micrographs of biofilms on pristine and NGs coated substrates.

A significant reduction in biofilm formation was observed on NGs coated samples compared to the pristine silicone material due to the intrinsic antimicrobial activity of PDDA-containing NGs, coupled to secondary inhibitory mechanisms such as quorum quenching inhibition. WNGs coating reduced the surface-associated biomass by approximately 60%. Incorporating ceragenin into the NGs further enhanced the biofilm inhibition, resulting in 80% and 90% biomass reduction and bacterial viability within the biofilms of and , respectively.

Biofilm distribution on the catheter material was examined using fluorescence microscopy and staining with a Live/Dead kit (Figure 8C). The CeNGs-coated samples displayed reduced and irregular biofilm formation, aligning with the quantitative biomass analysis. Additionally, SEM characterization revealed a markedly lower accumulation of both bacterial strains on the CeNGs-coated samples (Figure 8D).

To further validate the antimicrobial and antibiofilm performance of the coatings, an in vitro hydrodynamic model was designed to replicate the urinary environment^40^ (Figure 9A). Pristine and CeNGs coated catheters were inserted into artificial bladders inoculated with a mixed culture of most relevant , , , and , and artificial urine was continuously supplied at a controlled flow rate of 1 mL/min, mimicking the physiological urine output in adults (0.8–2 L per day).^41^ At the end of the 7 day experiment, the catheters were retrieved and analyzed to assess the coating’s efficacy in mitigating mixed-species biofilm formation in simulated bladder environment. The catheter coated with CeNGs demonstrated a marked reduction in both biofilm biomass and the viability of adherent bacteria on the lumen and outer surface (Figure 9B). This significant biofilm decrease indicates the high efficacy and durability of the coating under dynamic flow conditions.

**Figure 9:**
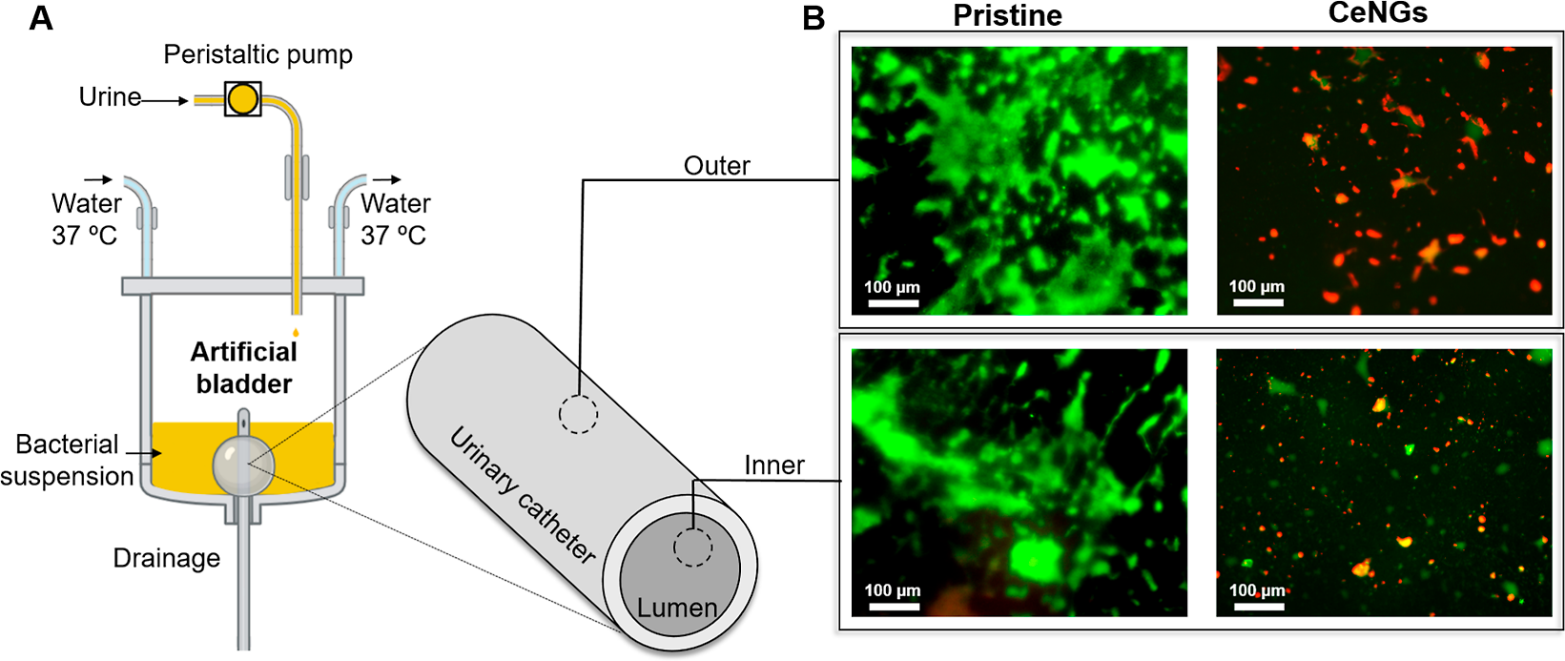
Antibiofilm properties of CeNGs coatings under dynamic conditions. (A) Schematic representation of the experimental setup used for the assessment of the biofilm formation under dynamic conditions. (B) Fluorescence micrographs showing live (green) and dead (red) bacteria on the lumen and external surface of catheters after exposure to a mixed bacteria culture for 7 days at 37 °C in dynamic conditions.

### 3.6 In vitro Biocompatibility

Biocompatibility is a fundamental requirement for indwelling medical devices. In this study, the biocompatibility of the NG-coated catheters was evaluated through in vitro cytotoxicity assays with human fibroblasts (BJ-5ta) and keratinocytes (HaCat) for 24 h (Figure 10A). No statistically significant differences were observed between the assessed samples, confirming the noncytotoxic nature of the coatings. Additionally, fluorescence microscopy images of cells stained using Live/Dead Kit showed a homogeneously distributed live cells with similar morphology for all samples (Figure 10B).

**Figure 10:**
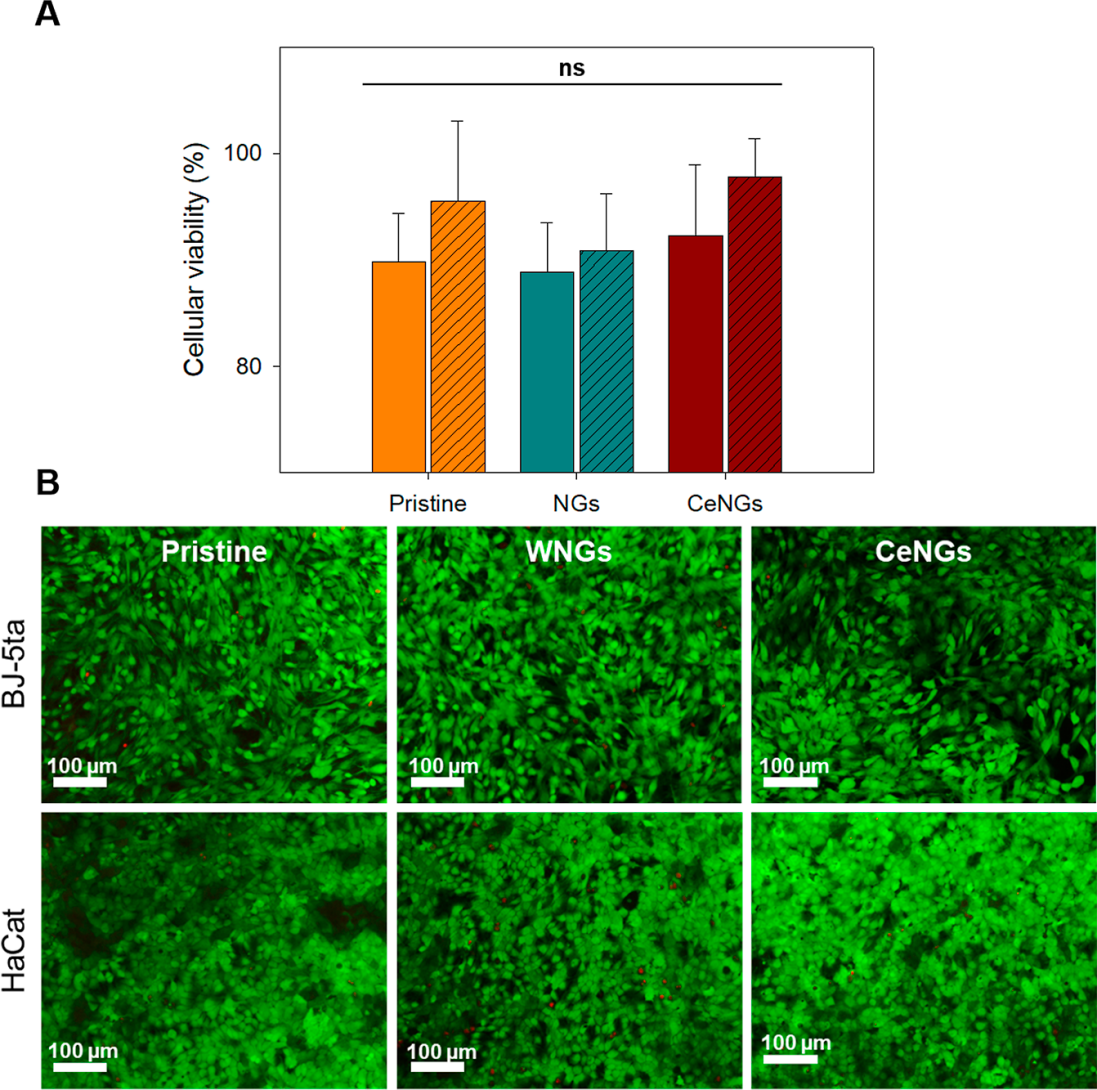
Viability of human fibroblast and keratinocyte cell lines upon exposure to pristine and coated silicone materials. Solid-colored bars represent fibroblast and diagonally hatched bars correspond to keratinocytes. (A) AlamarBlue and (B) Micrographs of samples stained with Live/Dead kit assays. The green and red fluorescence signals are overlaid (*n* = 3).

### 3.7 In vivo Studies

The hybrid CeNGs coatings were validated in vivo in catheterized rabbits during 10 days. During this period, the animals health was followed by hematological analyses of key cellular parameters (Table S1), which remained within the normal references limits until the end of the study. Biochemical profiling of blood samples further supported a general healthy state in both groups (Table S2), although elevated glucose levels indicative of physiological stress were observed, aligning with patterns previously observed in similar experimental settings.^42^

Urinalysis showed no statistically significant differences between the coated and uncoated groups (Table S3). At the beginning of the study, microbiological analysis of bladder-derived urine samples collected under sterile conditions confirmed the absence of bacteriuria. Following a 10 day catheterization, no pathogenic microorganisms were identified in animals fitted with coated catheters. In contrast, the control group bearing unmodified catheters displayed considerable microbial load exceeding 10^5^ CFU/mL, predominantly comprised of *Aerococcus* species, a bacterial genus frequently linked to CAUTIs.^43^ Following 10 days of catheterization with CeNG-coated catheters, the histological structure of both the urethra and kidneys remained unaltered, with no detectable abnormalities. Animals catheterized with pristine catheters exhibited no discernible renal tissue alterations, however, the urethral epithelium showed clear histopathological changes. These included an irregular and disorganized epithelial surface, luminal projections, epithelial hyperplasia, and signs of inflammation, likely attributable to infection and mechanical trauma associated with catheter withdrawal (Figure 11).

**Figure 11:**
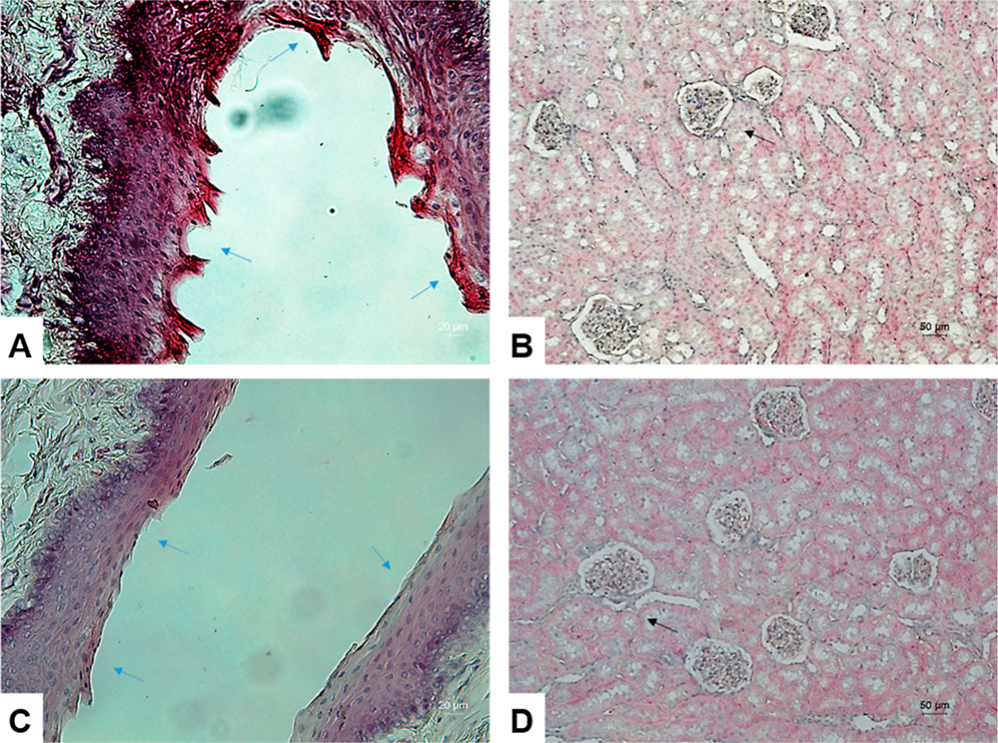
Histology of urethra (A,C) and kidneys (B,D) of male rabbits: (A,B) rabbits with control catheters; (C,D)­rabbits with CeNGs-coated catheters. Urethral epithelium (blue arrow). Renal corpuscles (black arrow).

The comprehensive assessment of clinical, histological, and microbiological data from the rabbit models confirmed that the CeNGs-coated catheters were biocompatible and effectively prevented catheter-associated urinary tract infections (CAUTIs) during the 10 day indwelling period. In contrast, the uncoated control catheters exhibited clear signs of bacterial colonisation and likely infection. Particularly concerning was the simultaneous presence of high levels of *Aerococcus* spp. in the control group, underscoring the rapid onset of infection during catheterization and highlighting the substantial risk of progression to more severe inflammation or the development of secondary pathologies.

## 4 Conclusion

In this study, a novel ultrasound-assisted, self-assembled NGs coating was developed on urinary catheters, offering a feasible and efficient strategy for prevention of biofilm formation, and potentially CAUTIs. PDDA-GA NGs loaded with ceragenin formed a robust coating with antimicrobial, antibiofilm, lubricating, and biocompatible properties, demonstrated several advantages over uncoated silicone catheters. The water-based sonochemical coating technology proved to be a straightforward and efficient approach for durable functionalization of catheters (maintaining antimicrobial and antibiofilm performance for over 7 days), without the need for prior substrate modification. Under hydrodynamic conditions, the coating reduced the bacterial colonisation on both lumen and outer surface of the catheter, as evidenced in an in vitro bladder model. Biofilm inhibition was attributed to contact-mediated bacterial killing and the controlled release of ceragenin, which suppressed bacterial proliferation, quorum sensing, and biofilm maturation. Biocompatibility studies confirmed the absence of cytotoxic effects associated with the CeNG coating. Finally, in vivo evaluations validated the antibacterial efficacy and safety of the coated catheters for clinical application. Taken together, these findings confirmed the effectiveness of the ultrasound-assisted CeNG coating. The synergistic combination of durable surface adhesion, lubricity, sustained antimicrobial release, potent antibiofilm activity, and excellent biocompatibility positions this approach as a promising strategy for the development of antibiofilm urinary catheters.

## Supplementary Material



## Notes

Blood cell counts and hematological profiles from rabbit in vivo studies (WBC, lymphocytes, monocytes, granulocytes, RBC, HGB, PLT); biochemical blood parameters including albumin, proteins, calcium, glucose, urea, enzymes, and creatinine levels; and detailed urinalysis data such as white blood cells, protein, pH, specific gravity, and metabolic indicators from coated and uncoated catheter groups (PDF)
