# Supplementary material for: Ceragenin Nanogel Coating Prevents Biofilms Formation on Urinary Catheters
Source: ACS Appl Mater Interfaces. 2025 Jul 22;17(31):44319–32. doi: 10.1021/acsami.5c13979 (PMC12332827; doi:10.1021/acsami.5c13979)
Supplement: Supplementary file 1 [file am5c13979_si_001.pdf]

Supporting information

Ceragenin nanogel coating prevents biofilms formation on urinary catheters

Antonio Puertas-Segura,[1] Antonio Laganá,[2] Garima Rathee,[1] Paul Savage,[3] Katerina Todorova,[4] Petar Dimitrov,[4] Iva Pashkuleva,[5, 6] Rui Luís Reis,[5, 6] Gianluca Ciardelli,[7] Tzanko Tzanov[1]*

[1] Grup de Biotecnologia Molecular i Industrial, Department of Chemical Engineering, Universitat Politècnica de Catalunya, Terrassa, Spain.
[2] Department of Biomedical and Dental Sciences and Morphofunctional Imaging, University of Messina, Messina, Italy.
[3] Department of Chemistry and Biochemistry, Brigham Young University, Provo, United States.
[4] Institute of Experimental Morphology, Pathology and Anthropology with Museum, Bulgarian Academy of Sciences, Geo Milev, Sofia, Bulgaria.
[5] 3B's Research Group−Biomaterials, Biodegradables and Biomimetics, University of Minho, Headquarters of the European Institute of Excellence on Tissue Engineering and Regenerative Medicine, 4608-017 Barco, Portugal.
[6] ICVS/3B's PT Government Associated Laboratory, Braga/Guimaraes, Portugal.
[7] Department of Mechanical and Aerospace Engineering, Politecnico di Torino, Torino, Italy.

*Author to whom correspondence should be addressed: tzanko.tzanov@upc.edu

Table S1. Blood parameters of each group (rabbits 1, 2, 3 non-coated and 4,5, and 6 with coated catheters) on the 10th day. Legend: White blood cell count (WBC), lymphocyte count (Lymph.), monocyte count (Mon.), granulocyte count (Gran.), red blood cell count (RBC), haemoglobin (HGB), platelet count (PLT).

**Parameters**	**Ref. Range**	**Rabbit 1**	**Rabbit 2**	**Rabbit 3**	**Rabbit 4**	**Rabbit 5**	**Rabbit 6**
WBC 10^9/L	5.2-13.5	9.7	11.2	10.7	13.2	11.7	12.9
Lymph. 10^9/L	3.2-9.0	3.2	3.2	3.9	6.0	5.9	5.6
Mon. 10^9/L	0.1-0.6	0.3	0.5	0.6	0.2	0.5	0.6
Gran. 10^9/L	2.0-7.5	6.2	7.5	6.2	7.0	5.6	6.7
RBC 10^{12}/L	5.00-7.60	5.4	5.6	5.2	5.6	6.0	5.8
HGB g/L	320-370	460	440	380	430	340	400
PLT 10^9/L	250-650	380	350	420	380	450	430

Table S2. Blood biochemical parameters of each group (rabbits 1, 2, 3 non-coated and 4,5, and 6 with coated catheters) on the 10th day. Legend: S_Albumin (ALB), total Protein (TP), globulin (GLO), calcium (Ca), glucose (Glu), blood urea nitrogen (BUN), phosphorus (P), amylase (AMY), cholesterol (CHOL), alanine transaminase (ALT), total bilirubin (TBIL), alkaline phosphatase (ALP), creatinine (CRE), creatine kinase (CK).

Parameters	Ref. Range	Rabbit 1	Rabbit 2	Rabbit 3	Rabbit 4	Rabbit 5	Rabbit 6
ALB	27-50 g/L	52.0	60.5	57.4	55.2	58.0	60.4
TP	49-71 g/L	32.9	45.0	39.6	33.4	40.4	30.9
GLO	15-33 g/L	19.1	15.5	17.8	21.8	17.6	29.5
Ca	2.2-3.9 mmol/L	3.5	3.0	3.4	2.4	2.5	3.0
GLU	5.5-8.2 mmol/L	H 12	H 13.5	H 12.9	H 14.0	H 13.6	H 13.4
BUN	10.1-17.1 mmol/L	10.6	11.01	14.0	10.8	12.02	12.7
P	1-2.2 mmol/L	1.5	2.0	1.2	1.5	2.19	1.2
AMY	212-424 U/L	335	285	339	320	238	290.8
CHOL	0.1-2 mmol/L	1.1	1.3	1.5	1.1	1.0	1.2
ALT	27.4-72.2 U/L	40.9	53.0	39.7	52.4	52.2	53.8
TBIL	2.6-17.1 μmol/L	78.6	80.5	90.0	69.4	86.0	100.3
ALP	12-96 U/L	2.6	3.4	3.9	5.1	4.7	3.9

CRE	74-1711 µmol/L	88.8	109.5	78.9	90.6	105.5	100.3
CK	58.6-175 U/L	80.5	70.9	76.4	85.3	77.3	79.6

Table S3. Urinalysis of representatives from each experimental group (rabbits 1, 2, 3 non-coated and 4,5, and 6 with coated catheters) on the 10th day (rabbits 1, 2, 3 and 4 with coated and rabbits 5 with non-coated catheters). Legend: White blood cells (WBC), ketone levels (KET), nitrites (NIT), urobilinogen (URO), bilirubin (BIL), glucose (GLU), protein levels (PRO), urine specific gravity (SG), ph measure of acidity or alkalinity, blood cells (BLD), urine vit. C (Vc), microalbuminuria (Ma), calcium (Ca), creatinine (CR).

**Parameters**	**Rabbit 1**	**Rabbit 2**	**Rabbit 3**	**Rabbit 4**	**Rabbit 5**	**Rabbit 6**
WBC cell/µL	0	0	0	0	0	0
KET mmol/L	0	0	0	0	0	0
NIT	---	---	---	---	---	---
URO	---	---	---	---	---	---
BIL µmol/L	0	0	0	0	0	0
GLU mmol/L	0	0	0	0	0	0
PRO g/L	0	0	0	0	0	0
SG	1.008	1.015	1.010	1.015	1.009	1.010
pH	7.8	8.0	7.9	8.0	8.0	8.0
BLD cell/uL	0	0	0	0	0	0
Vc mmol/L	0	0	0	0	0	0
MA mg/L	≤10	≤10	≤10	≤10	≤10	≤10
Ca mmol/L	7.5	7.5	7.5	7.5	7.5	7.5
CR mmol/L	≥26.4	≥26.4	≥26.4	≥26.4	≥26.4	≥26.4
